# An Unusual Case of Neonatal Hypotonia and Femur Fracture: Neuromuscular Variant of Glycogen Storage Disease Type IV

**DOI:** 10.3390/children10081375

**Published:** 2023-08-11

**Authors:** Handan Bezirganoglu, Kubra Adanur Saglam

**Affiliations:** 1 Division of Neonatology, Trabzon Kanuni Training and Research Hospital, Trabzon 61080, Türkiye; 2 Department of Medical Genetics, Karadeniz Technical University Medical Faculty, Trabzon 61080, Türkiye

**Keywords:** neonatal hypotonia, GSD-IV, neonatal fracture

## Abstract

Glycogen storage disease type IV (GSD IV) (OMIM #232500) is an autosomal recessive disorder caused by deficiency of the glycogen-branching enzyme. Here, we report a patient presenting with prematurity and severe hypotonia resulting from a complicated pregnancy with polyhydramnios. During her stay in the neonatal unit, the infant remained dependent on a ventilator, and her movements were mostly absent, except for occasional small movements of her fingers. A spontaneous fracture of femur shaft occurred in the postnatal fourth week. Whole-exome sequencing of DNA from the patient revealed a homozygous missense variant in the *GBE1* gene (c.1693C>T, p.Arg565Trp). The variation detected in the index case was also confirmed by Sanger sequencing in the patient and respective parents. This study showed that the neuromuscular subtypes of GSD-IV should be considered as a possible differential diagnosis in severe neonatal hypotonia cases.

## 1. Introduction 

Glycogen storage disease type IV (GSD-IV) (OMIM #232500) is a rare autosomal recessive inherited inborn error of carbohydrate metabolism caused by pathogenic variants in the *GBE1* gene. This gene encodes the glycogen-branching enzyme (GBE) that is responsible for adding branches to glycogen molecules. Mutations in the GBE lead to the formation of abnormal glycogen molecules. This abnormal polysaccharide molecules display poor solubility and tend to accumulate in various tissues, predominantly in the liver and muscle [1].

GSD-IV has been reported to occur in 1 in 600,000–800,000 births, with a remarkable heterogeneous clinical spectrum that varies in severity [2]. The age of presentation can also span from in utero through childhood to adult onset [3]. GSD-IV is classified into six different forms: fatal perinatal neuromuscular, congenital neuromuscular, classic progressive hepatic, non-progressive hepatic, juvenile neuromuscular, and adult onset [4]. The perinatal neuromuscular and congenital neuromuscular subtypes are extremely rare forms, with fewer than twenty genetically proven cases reported in the literature so far [5,6,7].

Here we report one of the rare fatal neonatal cases of GSD-IV determined by whole-exome sequencing and parenteral molecular testing. An infant with a family history of asphyxic birth in a previous sibling presents with severe hypotonia and poor respiratory effort at birth due to a pregnancy complicated by polyhydramnios. A femoral shaft fracture occurred during follow-up, and despite aggressive respiratory and physiotherapy support, the patient eventually died of cardiorespiratory failure.

## 2. Case

### 2.1. Initial Presentation 

A female infant delivered by emergency cesarean section because of decelerations during the nonstress test at 31 weeks gestational age and a birth weight of 1340 g. The pregnancy was complicated by polyhydramnios. Although the infant had a heart rate >100/minute at birth, she was severely hypotonic with markedly weak spontaneous respiration that required prompt intubation at the delivery room. She was transferred to the neonatal intensive care unit with initial diagnoses of prematurity, low birth weight, respiratory failure, and hypotonia. Physical examination of the infant revealed infrequent spontaneous breathing, lack of spontaneous eye opening or extremity movements, flaccid muscle tone, and the absence of neonatal reflexes. Nonetheless, normal deep tendon reflexes were observed, and no facial dysmorphisms, arthrogryposis, or organomegaly were noted. Upon follow-up examination, the patient’s activity status remained unchanged, and, despite low ventilator settings, she could not tolerate extubation attempts.

### 2.2. Family History 

**Figure 1:**
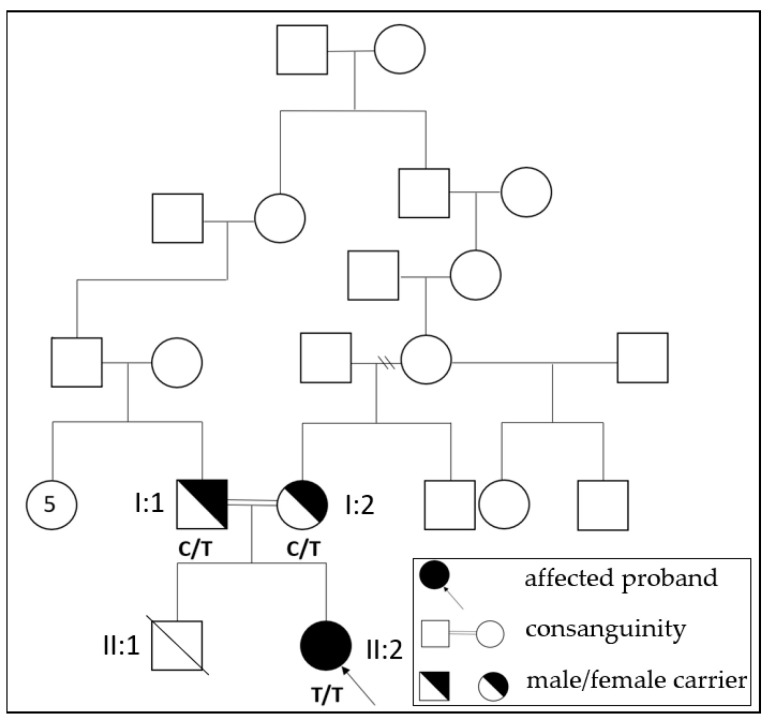
Pedigree of the family with the c.1693C>T (p.R565W) mutation in *GBE1.* Affected proband (II:2), consanguineous carrier mother (I:2) and father (I:1), ex sibling (II:1) shown in pedigree. (C: cytosine; T: thymine).

Her family history revealed a loss of a previous sibling due to birth asphyxia and severe hypotonia in the neonatal period. The previous pregnancy was complicated by polyhydramnios and reduced fetal movements. The infant born in that pregnancy required prolonged resuscitation at birth, including multiple doses of epinephrine and volume expansion. Unlike our present patient, the previous sibling exhibited mild arthrogryposis and contractures at birth and died on the postnatal 25th day due to multiorgan failure secondary to perinatal asphyxia. However, genetic testing was not performed in that case. The mother’s history revealed negative TORCH serology and no history of drug use, including benzodiazepines, during both pregnancies. Furthermore, the parents had consanguinity and had no living child (Figure 1). But there was no history of neuromuscular disorders in the family. 

### 2.3. Investigation

Initial arterial blood gas analysis did not indicate the presence of asphyxia, and cranial ultrasound revealed no structural abnormalities or intraventricular hemorrhage. Furthermore, an echocardiogram demonstrated no signs of congenital pathology or cardiomyopathy. An extended laboratory investigation was conducted to determine the underlying cause of severe hypotonia, which revealed elevated creatine kinase levels (1157 IU/L) and mild elevations in transaminase levels (ALT 62 IU/L and AST 82 IU/L, respectively). Blood gases, ammonia, lactate levels, glucose levels, serum amino acids, thyroid function tests, urine organic acids, and serology for intrauterine infection were all within normal limits. Abdominal ultrasound showed no pathology; moreover, brain magnetic resonance imaging on the postnatal 37th week did not reveal any structural abnormalities or morphological features suggestive of ischemia. Consequently, whole-exome sequencing was performed.

### 2.4. Genetic Testing 

The QIAamp DNA Blood Mini QIAcube Kit (Qiagen, Hilden, Germany) was used to obtain DNA from peripheral blood from the index case and parents. Whole-exome sequencing was performed on the index case using the Illumina NovaSeq6000 (Illumina Inc., San Diego, CA, USA) and the QIAseq^®^ Human Exome Kit (Qiagen, Hilden, Germany). FastQC software (version 1.0.0) was used to assess read quality, and poor-quality reads were eliminated using Trimmomatic (version 0.38). Sequence reads were aligned to the Human Reference Genome (hg19/GRCh37) using BWA-MEM (version 0.7.17) and converted into BAM files via SAMtools (version 1.3). Filters were applied to assess the pathogenicity of the resulting variants: (1) nonsynonymous, missense, nonsense, frameshift, splice-site, no-stop, no-start, and inDels variants in all protein-coding genes; (2) intronic variants affecting synonymous or splice region; (3) variants with a frequency of <1.0% in population studies; and (4) variant frequencies of 20–100% were included in the evaluation. The variants obtained after the filtering steps were evaluated according to the patient’s phenotype. In silico prediction tools (SIFT, PROVEAN, GERP, CADD, PolyPhen2 and MutationTaster), population frequencies (Genome Aggregation Database (gnomAD)), segregation analysis and American College of Medical Genetics and Genomics (ACMG) criteria were used to evaluate the pathogenicity of the variants. The variant was visualized with Integrative Genomics Viewer (IGV). The HOPE Web server (http://www3.cmbi.umcn.nl/hope/, accessed on 29 June 2023) was used to analyze the structural effects of the obtained variant on the protein sequence. The variant identified as pathogenic in ClinVar and/or Human Genome Mutation Database Professional (HGMD^®^ Pro) was considered to explain the phenotype. The variant, which is thought to explain the current clinical state, was investigated by Sanger sequencing in the index case and respective parents. The primers for exon 13 of the *GBE1* gene used in Sanger sequencing were forward 5’-CCTGTGCTAGACCGTGCTAT-3’ and reverse 5’-TGGTACAGGACACTAGACACT-3’.

### 2.5. Genetic Analysis and Functional Interpretation

**Figure 2:**
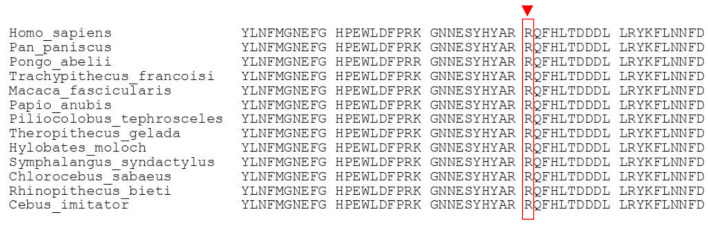
Amino acid sequence alignment of GBE from different species. Red box shows arginine, the amino acid at codon 565 of the GBE protein, which is conserved across different species.

**Figure 3:**
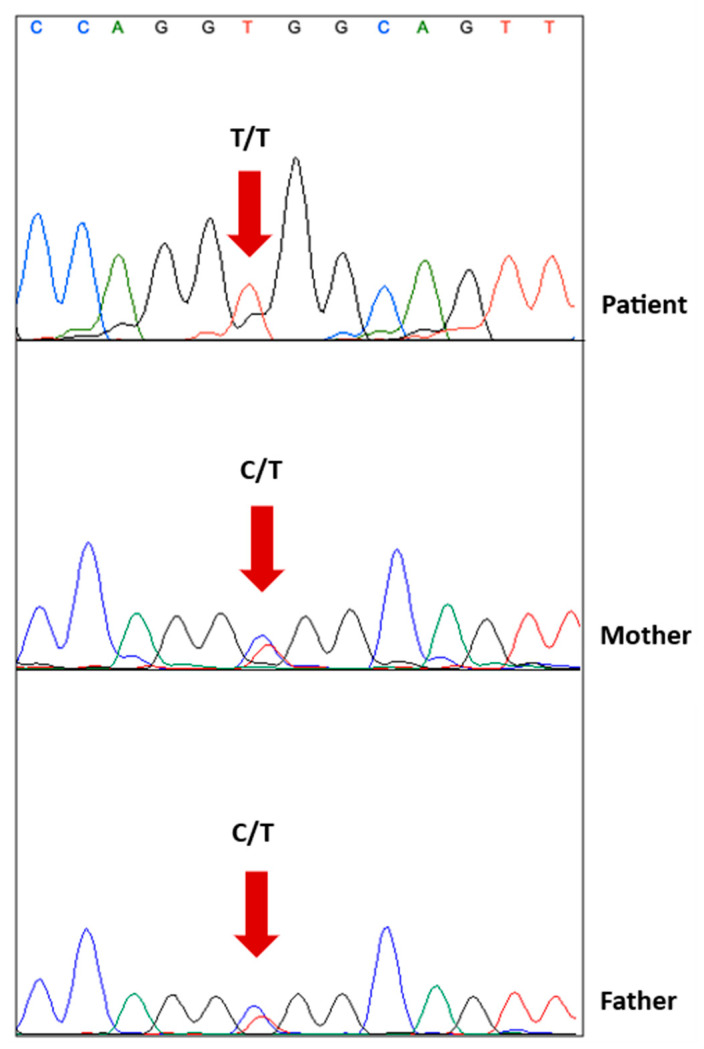
Verification of the c.1693C>T variant in the *GBE1* gene. Sanger sequencing results showing that the patient was homozygous for c.1693C>T (**top**), and also a heterozygosity of the locus was detected in the patient’s mother (**middle**) and his father (**bottom**). (C: cytosine; T: thymine).

In the whole-exome sequencing, a homozygous missense variation was detected in the *GBE1* gene (NM_000158.4: c.1693C>T, p.R565W). This specific mutation (c.1693C>T) has been previously reported as a disease-causing mutation in the HGMD database (CM202873). Moreover, the ClinVar database has conflicting interpretations regarding its pathogenicity, with some entries suggesting it is a variant that is likely pathogenic (RCV001329659.1, RCV000855462.1), while others indicate uncertain significance (RCV001829394.1, RCV002528222.1) (source: http://www.ncbi.nlm.nih.gov/clinvar/, accessed on 29 June 2023). In both the HGMD and ClinVar databases, this variation has been associated with the fetal akinesia phenotype [8]. Additionally, in the ClinVar database, it has been linked to the polyglucosan body disease, adult form (OMIM #263570) (RCV001329659.1). Furthermore, this variation is rare in the gnomAD database, with a population frequency of 0.000008901, and it has never been reported as homozygous before. Its presence is also evolutionarily conserved across species (Figure 2). According to the ACMG criteria (PM2, PM3, PP3, PP5), this *GBE1* variant was evaluated as likely pathogenic and was considered to explain the patient’s clinical condition. To further validate the finding, the variation detected in the index case was confirmed by Sanger sequencing in both the mother and the father (Figure 3).

This *GBE1* variant results in the substitution of arginine, a polar and basic amino acid, by tryptophan, an apolar and aromatic amino acid [9]. In the wild-type peptide, the arginine residue at position 565 hydrogen bonds with methionine at position 539 [10]. The difference in size and hydrophobicity between the wild-type and mutant peptide prevents the tryptophan from being in the correct position to make the same hydrogen bonding that the wild-type peptide makes [10]. The *GBE1* c.1693C>T variation is located in exon 13, which is consistent with previous findings that disease-causing mutations were predominantly clustered in the catalytic region of the GBE protein and in exons 12 and 13 of the *GBE1* gene [11,12].

### 2.6. Follow-Up and Outcome 

**Figure 4:**
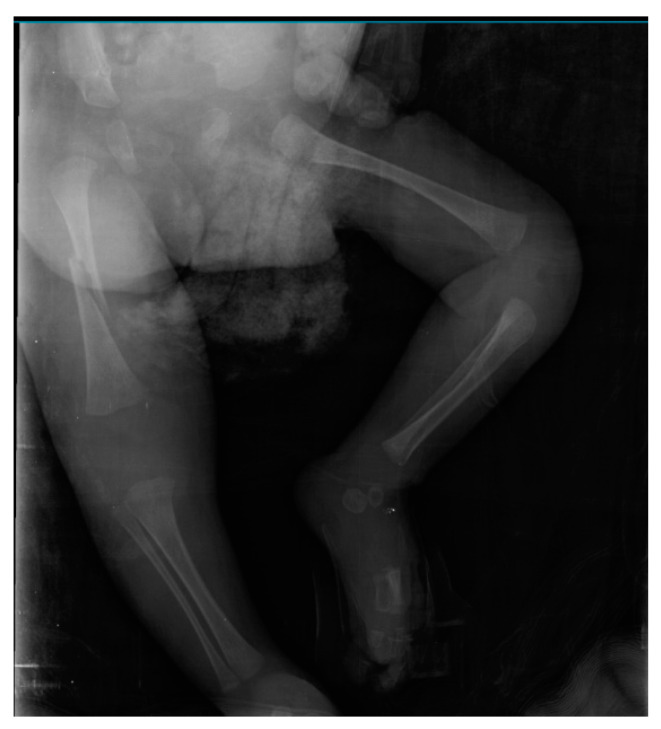
Oblique fracture of right femoral shaft of the patient.

During her stay in the neonatal unit, the infant remained dependent on a ventilator and required increasing levels of oxygen. Additionally, she developed a bronchopulmonary infection and received antibiotics for treatment. Her movements were mostly absent, except for occasional small movements of her fingers. On the postnatal 28th day, she was noticed to have a spontaneous fracture of the right femur shaft, which was managed by splinting (Figure 4). There was no known history of trauma. Her biochemical analysis showed normal levels of calcium, magnesium, phosphorus, parathyroid hormone, and alkaline phosphatase. Nutrition review showed adequate vitamin D intake. On the postnatal 60th day, a tracheostomy and gastrostomy were performed due to prolonged intubation. The infant died on the postnatal 100th day after clinical deterioration and cardiorespiratory failure unresponsive to treatment. The family did not permit muscle biopsy or autopsy.

## 3. Discussion

We described a patient presenting with prematurity and severe hypotonia resulting from a pregnancy complicated by polyhydramnios. Clinical presentation, a history of neonatal sibling demise due to asphyxia and severe hypotonia, and the molecular finding of a pathogenic variant *GBE1* gene (NM_000158.3) were consistent with the perinatal/congenital neuromuscular subtype of GSD-IV. Whole-exome sequencing of DNA from the patient revealed homozygous missense variant in the *GBE1* gene (c.1693C>T, p.Arg565Trp). To our knowledge, this is the first reported case diagnosed with the perinatal neuromuscular subtype of GSD-IV in which spontaneous fracture of long bones occurred during the follow-up period.

There is a scarcity of literature on perinatal neuromuscular and congenital neuromuscular subtypes of GSD-IV, with some authors having combined these two distinct subtypes into a single group [13]. Previous reported cases in the literature did not establish a clear genotype–phenotype correlation [4]. However, it is thought that patients with two null variants or at least one protein-truncating mutation tend to have a more severe neuromuscular phenotype [12]. Nevertheless, as observed in our case, it has been documented that two missense mutations can also lead to early-onset neuromuscular phenotypes of GSD-IV [14,15,16]. Bruno et al. reported two affected siblings born to consanguineous Syrian parents with a homozygous missense mutation in exon 13 of the *GBE1* gene, exhibiting the fetal akinesia deformation sequence (FADS) phenotype and GSD-IV [17]. This supports the fact that missense mutations in exon 13 as described in our patient can cause a severe neuromuscular phenotype that may be fatal in the neonatal period. However, the impact of this variation on enzyme activity should be further validated through functional studies. The lack of functional studies on the *GBE1* gene c.1693C>T (p.R565W) mutation was a limitation of this study. 

Our patient’s mutation, c.1693C>T, p.Arg565Trp in the *GBE1* gene, has been reported in only one case in the literature [8]. That patient presented with muscular hypotonia, pulmonary hypoplasia, joint contractures, and dysmorphic features. The pregnancy was complicated by polyhydramnios and hydrops fetalis in that case. Although the results could not be confirmed, we speculated that the first child of the present family, who presented with asphyxia and severe hypotonia, may also have the same pathogenic variant of the *GBE1* gene. Clinical variability among GSD-IV cases has been attributed to the specific type and location of the mutations within the *GBE1* gene and their resulting impact on the structure and function of the protein [3]. However, despite having an identical mutation, the first described patient [8] had a more severe clinical presentation during the perinatal period compared to our patient and her sibling. For our patient, the timing of symptom onset was most consistent with the perinatal neuromuscular subtype. Nonetheless, the overall clinical course more closely resembled the congenital neuromuscular subtype. This finding supports the suggestion that distinguishing these two subtypes is challenging and variations among GSD-IV patients exist along a continuum [4].

Our patient had elevated creatine kinase levels. According to a recent review, patients with both the severe and lethal neuromuscular forms, as well as the less severe neuromuscular form of GSD-IV, can exhibit significantly elevated CK levels [18]. The present case experienced a spontaneous femoral shaft fracture during the follow-up period. Previous studies have demonstrated that congenital and neonatal fractures are observed in disorders associated with reduced fetal movement, such as spinal muscular atrophy and congenital myopathies [19,20]. It is believed that decreased mechanical loading on developing bones, muscle atrophy, and joint deformities resulting from fetal akinesia and immobilization in the neonatal period contribute to bone fragility and predispose individuals to fractures during the intrauterine and neonatal periods [21]. In our patient, the bone fracture occurred during the postnatal 4th week following prolonged immobilization. This highlights the importance of considering neuromuscular subtypes of GSD-IV in the differential diagnosis of neonatal fractures, especially when severe hypotonia is present.

## 4. Conclusions

In neonates presenting with hypotonia and pregnancies complicated by polyhydramnios and decreased fetal movement, it is essential to consider the neuromuscular subtypes of GSD-IV as a possible differential diagnosis. Moreover, contrary to previous generalizations, this case illustrates that certain missense pathogenic variants in *GBE1* can also cause severe congenital neuromuscular subtypes of GSD-IV.

## Data Availability

Not applicable.
